# A structure and knowledge-based combinatorial approach to engineering universal scFv antibodies against influenza M2 protein

**DOI:** 10.1186/s12929-023-00950-2

**Published:** 2023-07-25

**Authors:** Ujjwal Kumar, Priya Goyal, Zaid K. Madni, Kajal Kamble, Vineet Gaur, Maitreyi S. Rajala, Dinakar M. Salunke

**Affiliations:** 1 International Centre for Genetic Engineering and Biotechnology, Aruna Asaf Ali Marg, New Delhi, 110067 India; 2 Jawaharlal Nehru University, New Delhi, Delhi, 110067 India; 3 National Institute of Immunology, Aruna Asaf Ali Marg, New Delhi, 110067 India; 4 National Institute of Plant Genome Research, Aruna Asaf Ali Marg, New Delhi, 110067 India

**Keywords:** Influenza virus, M2 ion channel protein and M2e dimerization, Naive human antibody scFv library, Co-crystallization, Virus regression

## Abstract

**Background:**

The influenza virus enters the host via hemagglutinin protein binding to cell surface sialic acid. Receptor-mediated endocytosis is followed by viral nucleocapsid uncoating for replication aided by the transmembrane viral M2 proton ion channel. M2 ectodomain (M2e) is a potential universal candidate for monoclonal antibody therapy owing to its conserved nature across influenza virus subtypes and its importance in viral propagation.

**Methods:**

The phage-displayed naive human antibody libraries were screened against the short stretch of the N-terminal 10-mer peptide (SLLTEVETPI) of the M2e. ELISA, BLI, and flow cytometry assays were used to examine scFv binding to M2e epitopes. The scFv crystal structures were determined to examine the nature of the interactions. The potencies of the scFvs against the influenza virus were demonstrated by real-time PCR and confocal microscopy imaging.

**Results:**

The four unique scFv clones were obtained from the scFv phage-display antibody libraries and shown to exhibit binding with the 10-mer conserved part of the M2e and with full-length M2 protein expressed on the HEK293T cells. The crystal structure of scFv AU1 with M2e peptide showed the peptide as a dimer in the parallel beta-sheet conformation bound at the interface of two scFv CDRs. The scFv AU1 significantly restricted the release of H1N1 virus progeny from the infected A549 cells.

**Conclusion:**

This structural and biochemical study showcased the binding of antibody scFv molecules with M2e peptide dimer, providing the structural insights for the function effect in terms of recognizing and restricting the release of new viral particles from an infected host cell.

**Supplementary Information:**

The online version contains supplementary material available at 10.1186/s12929-023-00950-2.

## Background

Influenza, commonly known as flu, infects the respiratory system and is the primary cause of high morbidity and mortality worldwide [1]. Influenza viruses are among the fast-mutating viruses having segmented negative-strand RNA genomes enveloped within a host-derived lipid membrane. Fast-mutating viruses tend to incorporate genetic mutations into the hemagglutinin and neuraminidase glycoproteins on the viral surface to escape immune surveillance without affecting their normal functions. These mutations often lead to the emergence of new variants of the viruses with high virulence and infectivity rates [2]. Influenza viruses infect various hosts like humans, pigs, and birds. It has also been reported that they can cross the species barrier by host adaptation and the reassortment of gene segments leading to sustained transmission and creating novel viral strains [3]. Emergence and re-emergence of these new strains, which are naive for the human population, result in epidemic and pandemic outbreaks [4]. Live attenuated and inactivated influenza vaccines have traditionally been administered to prevent flu. In addition, a synthetic trivalent influenza vaccine comprising an H1N1, an H3N2, and an influenza B virus strain is formulated and given annually [5]. These vaccines are reformulated every year due to random antigenic shifts and drift in their surface glycoproteins [6]. To counter novel influenza strains naive to humans, there is always a need for improved vaccines that can give cross-reactive immunity against different strains or generate antibodies against the epitopes, which can bind and neutralize all strains.

A suitable candidate to counter influenza viruses through one universal vaccine is the surface protein, matrix protein 2 (M2), which is a relatively conserved protein across all the strains [7, 8]. The M2 is a proton-selective ion channel, and it plays a pivotal role in the uncoating of the viral core during entry and viral assembly [9]. The viral core comprises of M1 matrix protein 1 (M1), nucleoprotein, three subunits of RNA polymerase, and non-structural viral proteins. After the fusion of the virus envelope to the endosomal host membrane, the M2 ion channel gets activated due to the low pH of the endosome and initiates transporting protons across the membrane [10]. The acidic environment of virion weakens the interaction between the M1 matrix protein and the viral ribonucleoproteins, which leads to the release of viral genetic material into the host cell cytoplasm. Subsequently, the viral genetic material is imported to the nucleus to initiate the replication [11].

Each M2 monomer is a 97 amino acid long transmembrane protein comprising of an outer M2e ectodomain (1–24), a middle α- helical transmembrane region (25–46), and an inner cytoplasmic tail (47–97). The M2 exhibits a pH-dependent oligomeric state existing as a dimer at neutral pH and organizing as a tetramer at acidic pH [12]. The M2 homotetramer comprises of four parallel M2 with two disulphide linked dimers held together by noncovalent interactions [13]. Interestingly, the disulphide bonds are not essential for the maintenance of the oligomeric state [14]. The M2e has low variability in all influenza virus strains and is accessible to antibodies and therefore is a central theme in several studies [15, 16]. Some monoclonal antibodies (e.g., 14C2) raised against the M2 protein could not block the virus’s attachment with the host cells while limiting the virus load [17]. At the same time, some showed protection in the mouse model against a lethal dose of the influenza virus [18, 19]. Interestingly, some monoclonal antibodies bind exclusively to the dimeric M2e, while others can bind to both monomeric and dimeric forms of M2e [20]. Such interactions of M2e peptide with antibody and its dimerization capability in the context of inhibiting virulence have not been studied at the structural level earlier.

Owing to being solvent-exposed and having relatively high sequence conservation, we aimed to generate monoclonal antibodies against the M2e domain of the M2 protein and explore possible neutralization of the virus by the antibodies. A short conserved N-terminal region of the M2e (SLLTEVETPI) was narrowed down based on comparative sequence analysis for screening human single-chain variable fragment (scFv) libraries. Four unique scFvs were obtained that were expressed and purified for further biochemical, cell-based, and structural studies. Our structural analysis provided insights into the mechanism of binding of scFv AU1 with the M2e epitope. Interestingly, we found that the scFv-bound M2e peptide exists in the dimeric form with scFv through complementarity determining regions (CDRs) at the interface of two monomers. Furthermore, one of the scFvs (AU1) significantly inhibited the release of viral progeny from infected cells. We provide a model to explain the mechanism of M2 inhibition during the viral propagation from the infected cells by binding to the M2 protein in its native state.

## Material and methods

### Selection of peptide

All 66,692 M2 protein sequences of influenza viruses deposited up to 2019 were extracted from the flu database (http://www.fludb.org). After the multiple sequence alignment in Clustal W [21], we analyzed the mutational frequency occurrence of the N-terminal 24 residues in the M2 protein. We shifted the frame by one amino acid to get partial overlap with another peptide which was variable just to explore possibility of cross-reactive antibodies. Based on this analysis, the 10-mer peptide epitope (U1-SLLTEVETPI) from the N-terminal of the M2 protein was selected. U1 peptides were purchased from BIO-HELIX. All these peptides have the first lysine residue for conjugation to bovine serum albumin (BSA) (Sigma) using glutaraldehyde (Sigma).

### Phage display scFv library screening against the N-terminal epitopes

Human scFv libraries (Tom I + J), KM13 (helper phage), TG1 (*E. coli*), and HB2151 (TAG stop codon suppressor) were procured from MRC laboratory, Center for Protein Engineering (Cambridge UK). Strategies for the biopanning method against the BSA conjugated peptide were followed as reported in an earlier publication [22]. Briefly, round bottom PolySorp or MaxiSorp Immuno tubes (Nunc, Rochester, NY) were coated with 4 ml of BSA conjugated U1 peptide and only BSA in phosphate buffer saline (PBS) at 4 ^ο^C overnight. After washing the tubes three times with PBS, they were blocked with 2 % skimmed milk powder (MPBS) for 1 h at room temperature (RT). To remove the BSA-specific phages, scFv phage libraries (10^12^ in 2 % MPBS) were incubated with BSA-coated immuno tubes for 1 h at room temperature. Subsequently, phage libraries were incubated with BSA-conjugated U1 peptide for 2 h at RT. To remove the non-specific phages in BSA conjugated U1 peptide, they were washed with 0.1 % Tween 20 in PBS (10 times in the first biopanning, 20 and 30 times in the second and third biopanning, respectively). Only persistently bound phages remained during the washing, and they were eluted with incubation of free U1 peptide in 1 mg/ml concentration for 30 min at RT for every biopanning round. TG1 *E. coli* were infected with eluted phages and plated on 2x TY media plates. The next day, TG1 *E. coli* scraped with media and amplified till the O.D. reached 0.4 nm. Afterward, KM13 helper phages were infected with TG1 *E. coli*, which helped to make more selected scFv phages for the next round. This cycle was repeated 4 times against the U1 peptide.

### Polyclonal and monoclonal phage enzyme-linked immunosorbent assay (ELISA)

For the polyclonal and monoclonal phages, ELISA was also adopted as previously reported [22]. Briefly, BSA conjugated U1 peptide (100 mg/ml) and BSA as negative control were coated on immunosorbent plate wells at 4^o^ C overnight. Purified phages (10^12^ in PBS) were added into the well for 2 h at RT. After the incubation, immunosorbent plates were well-washed 3 times with the PBST (PBS + 0.1 % Tween 20). It was incubated with secondary antibody (HRP-conjugated anti-M13 antibody GE Healthcare Cat# 27,942,101) in 1:5000 dilution for 1 h at 37^o^ C and was developed by the ortho-phenylenediamine (OPD) (Sigma) and H_2_O_2_ as substrate. A microplate reader was used to measure the intensity of color at 490 nm.

In the monoclonal ELISA, we picked individual 72 colonies from the bacterial plate in the last selection round, purified the individual phages, and followed the same steps described above for the ELISA. After the monoclonal ELISA, high-binder phages were selected for DNA sequencing (Macrogen. Inc).

### Cloning, expression, and purification of scFv

Each unique clone of scFv in the pIT2 phagemid vector isolated from TG1 was digested with NcoI and NotI restriction enzymes (Thermo fast digestion) and cloned into the pET22b vector (EMD Biosciences) for the scFv expression. It was transformed into the BL21 *E. coli* strain, induced by 1 mM IPTG at 1.5 nm O.D._600_ and grown overnight at 18^o^ C. For the periplasmic extraction, two periplasmic extraction buffers (PEB) were used. PEB1 buffer composition was 100 mM Tris pH 8.0, 1 mM EDTA, and 20 % sucrose, and PEB2 buffer contained 5 mM MgCl_2_ in deionized water [23]. Firstly, the bacterial pellet was dissolved in PEB1 buffer and incubated for 45 min on ice. The dissolved pellet was centrifuged at 16,000x*g* for 15 min, and the supernatant was collected. After that, the remaining pellet was dissolved into the PEB2 buffer and incubated at ice for 20 min. The supernatant was collected after centrifugation and mixed with PEB1 buffer. For the Ni-NTA purification, the supernatant was loaded on the Ni-NTA column (GE Healthcare) and eluted with different concentrations of imidazole. Eluted scFv was dialyzed against the 50 mM Tris pH 8.0 buffer containing 100 mM NaCl. Size exclusion chromatography of scFv was performed using Sepharose S-75 column (GE Healthcare).

### Soluble scFv ELISA and bio-layer interferometry (BLI)

In soluble scFv ELISA, BSA conjugated U1 peptide epitope (2 µg/ml) in PBS was coated in the well of the immunosorbent plate and incubated overnight at 4^o^ C. 2 % skimmed milk was used to block the plate for 1 h. Purified scFv at different concentrations (highest 100 µg/ml) was incubated for 2 h. All experiments were performed on Octet RED96e BLI equipment for antigen-antibody binding analyses. We defined the assay plates column in the BLI software and assigned the 96 well plates column as the baseline for 60 s, the association for 120 s, and the dissociation for 300 s. scFv as a binding partner used in decreasing concentrations with the 1/3 dilution like as 20 mM, 6 mM, 2 mM, 0.7 mM, and 0.2 mM. 10 mM glycine buffer at pH 2.5 in Milli-Q water was used for the degeneration of Super Streptavidin (SSA) sensors for each concentration, and neutralization of the SSA sensor was achieved in PBS. We used biotinylated BSA conjugated peptide (100 µg/ml) in PBS buffer to immobilize SSA biosensors at 25^o^ C with shaking at 1000 rpm. Data acquisition and analysis were done on the octet96e software version 10.0.01 [24].

### Flow cytometry

Wild type Myc-DDK-tagged ORF clone of viral ORF for matrix protein 2 [Influenza A virus (A/New York/392/2004(H3N2))] plasmid was purchased (Cat: VC102459). M2 plasmid was transfected into HEK293T (procured from ATCC) at 1 µg concentration in a 60 mm dish using lipofectamine 2000 (Life Technologies). Cells were harvested with chilled 50 mM ethylenediaminetetraacetic acid (EDTA) in PBS after 48 h. After this step, cells were washed 3 times with 3 % fetal bovine serum (FBS) in PBS and incubated for 30 min for blocking. Primary scFv antibody was added and incubated for 1 h at 4^o^ C. Rabbit anti-histidine secondary antibody (His-Tag (D3I1O) XP® Rabbit mAb #12,698, CST ) was used for staining the cells and incubated for 20 min at RT. Stained cells were analyzed on flow cytometer using relevant software (BD Biosciences).

### Crystallization, data collection, and structure determination

We explored crystallization conditions for scFvs and their complexes with the peptide epitope using a wide range of crystallization Hampton Research (Cat no. HR2-130, HR2-452, HR2-086) screens. For the complex crystals, the extended peptide (SLLEVETEVPIRNEWG) was dissolved into the 100 % DMSO and incubated with scFv in a 1:5 ratio for 1 h at 20^o^ C. Afterward, it was spun at 13,000 rpm for 10 min, and the hanging drop vapor diffusion experiments were set up for crystallization. Crystals grew within 7 days and frozen in liquid nitrogen.

Crystal diffraction data were collected at the ESRF beamline ID30B, equipped with a modified microdiffractometer (MD2-S), and Elettra beamline XDR2, equipped with a Dectris Pilatus 6 M detector. Recorded images were processed with the autoPROC and HKL3000 softwares [25, 26]. The Phenix software 1.19.2 [27] was used for performing molecular replacement and refinement using initial model 7YUE. A subset of 5 % reflections was set aside for Rfree calculation during model refinement. Map-fitting tool Coot-0.9.81 was used for structure analysis and comparison [28]. PyMOL Molecular Graphics System, Version 2.5 and Schrödinger, LLC were used for structure visualization, analysis and images preparation. All the data statistics were provided in Additional file 1: Table S1, for the purpose of internal consistancy.

### Molecular dynamics (MD) simulation

Molecular dynamics simulations were performed using the Schrodinger suite in the Desmond module [29]. Firstly, we prepared the scFv and peptide complex for energy minimization in the protein preparation wizard. The periodic boundary was created around the protein complex in a shape of an orthorhombic box which was saturated with water molecules using the TIP3P solvation model. Salt was added at a concentration of 150 mM for neutralization. To run the final MD simulation, default parameters like the NPT ensemble system (isobaric-isothermal condition), the constant temperature at 300 K, and the constant pressure at 1.01325 bar were set up. An MD simulation run was carried out under the OPSL4 force field for 500 ns (nanoseconds), and time was recorded at 2 frames per second. The simulation interaction module was used after the completion of MD simulation run for the analysis of the interactions between the complex, and the graph was plotted with respect to root mean square deviation (RMSD) values for the backbone. The same procedure was carried out for MD simulations of the peptide dimer without scFvs, and the graph was plotted for the peptides backbone atoms.

### Cell line and viruses

Lung carcinoma epithelial (A549) and Madin-Darby canine kidney (MDCK) cells were used for in vitro study. These cell lines were acquired from National Centre for Cell Science (NCCS), Pune. A549 cell line was used for real-time PCR analysis, while MDCK cells were used for viral propagation and to observe cytopathogenic effect (CPE). Cells were cultured in high glucose Dulbecco’s Modified Eagle Medium (DMEM) supplemented with 10 % FBS and 1X antibiotic solution (containing Penicillin and Streptomycin) in a humidified air chamber at 37 °C with 5 % CO_2_. All the cell culturing reagents were purchased from Hi-media. Influenza A virus of 2009 H1N1 pandemic isolated from patients at AIIMS, New Delhi, was used in the study.

### Virus propagation

Influenza virus stock was propagated for experimental studies. The MDCK cells were grown in the media supplemented with 1 µg/ml TPCK (Sigma), 20 mM HEPES (pH 7.2) (Sigma), 1X antibiotics, and 0.2 % BSA (Sigma) at 37 °C with 5 % CO_2_ in a humidified chamber. MDCK cells were seeded at a density of 1 × 10^4^. After attending the 80–90 % confluency, cells were washed twice with PBS and once with DMEM supplemented with 1 µg/ml TPCK (Sigma), 20 mM HEPES (pH 7.2) (Sigma), and 1X antibiotic solution. Virus inoculum was diluted in the above-supplemented medium at an multiplicity of infection (MOI) of 2 and incubated for 1 h at 37 ^o^C in CO_2_ incubator. MDCK cells were also mock-infected with supplemented media containing no virus. After 1 h viral adsorption, media were changed with fresh DMEM medium supplemented with 1 µg/ml TPCK (Sigma), 20 mM HEPES (pH 7.2) (Sigma), 1X antibiotics and 0.2 % BSA (Sigma). The infected cells were further incubated at the above-described conditions till 70–80 % of the cell monolayer exhibited CPE. The infected cells were harvested and freeze-thawed thrice, and the supernatant containing the virus was stored at − 80 °C in aliquots. The viral titre was measured by TCID50 assay.

### Quantitative real-time PCR

A549 cells were grown in 6-well plates (Corning) and infected with the influenza A virus. Post viral adsorption, cells were treated with different scFv (100 µg/ml) antibodies for 8 h. Cell monolayer and supernatant (Media) were harvested separately after 8hrs post-infection, and total RNA was isolated using TRIzol Reagent LS (Sigma). Verso cDNA Synthesis Kit (Cat no: AB1453A) was used to synthesize cDNA from 500 ng of RNA according to the manufacturer’s instructions and then subjected to real-time PCR using KAPA SYBR® FAST qPCR Master Mix (2X) Kit (Cat no: KK4602) to measure the level of the viral transcript, i.e., nucleoprotein (NP) using specific primers; NP-FP: 5'- AAGCAGATACTGGGCCATAAGG-3’ NP-RP: 5'- GAGAATGTAGGCTGCACACTGATC-3’. GAPDH-FP: 5’- TGCACCACCAACTGCTTAGC-3’; RP: 5’- GGCATGGACTGTGGTCATGAG-3’ glyceraldehyde 3-phosphate dehydrogenase (GAPDH) was used as an internal control to normalize the amount of RNA used in the reactions for cDNA synthesis. PCR reaction cycles were performed by CFX96 Touch Real-Time PCR Detection System (Bio-Rad). Comparison of NP expression between mock and viral infected cells was done by calculating the ‘n’ fold difference in mRNA abundance using relative quantification, 2 -ΔΔCT method. Where ΔCT = CT of target gene − CT of a control gene. ΔΔCT = ΔCT of virus − ΔCT of Mock. NP gene expression in mock infected cells was taken as a control, and the expression level was considered one for comparison. Reactions were performed in triplicate. Error bars reflect the standard error of the mean from three independent experiments for the influenza virus RNA quantitation studies.

### Cytopathogenic effect (CPE)

Seed MDCK cells in complete growth medium into 6-well cell culture plates at a density of 10^4^ cells per well and incubate the plates at 37˚C with 5 % CO2 until the cells reach 80–90 % confluency. After that, the cell monolayer was infected with the influenza virus A at an MOI 5 for 1 h. Cells were washed with PBS and change the medium after 1 h to remove the surface attached viruses. After the viral infections in cells, scFv AU1 incubated with infected cells in the medium at 37˚C with 5 % CO2 for 48 h. Using an Olympus CKX41 light inverted microscope, the visual evaluation of virus-induced CPE was checked after 48 h (Olympus, Tokyo, Japan).

### Immunofluorescence assay (IFA)

A549 cells were grown on 6-well plate containing 12 mm cover slips to 80–90 % confluency. Cells were infected with influenza A virus at an MOI of 2 at 1 h time point and then processed for IFA. After 24 h post infection, cells were washed twice with PBS and then fixed with chilled acetone at − 20 °C for 20 min. Acetone was removed and fixed cells were washed with PBS and subjected to IFA staining using primary antibody against viral M1 and incubated overnight at 4 °C in moist chamber. Next day, coverslips were washed with chilled PBS thrice and then probed with anti-rabbit FITC (green) conjugated secondary antibody (Sigma-Aldrich) at 37 °C for 1 h. After three washes nuclei were stained with 2.5 µg/ml of Hoechst stain for 10 min at RT. Cover slips were mounted on glass slides and the protein expression was visualized under Confocal Laser Scanning Microscope (Olympus FluoviewTM – FV3000) equipped with HeNe laser (488 nm), HeNe laser (640 nm) and pulse diode laser (405 nm). Images were acquired with GaAsP 100X NA: 1.45 oil immersion objective using CELLSENS 3.2 software.

### Statistical analysis

Student’s t-test and one-way analysis of variance (ANOVA) with Bonferroni’s correction for multiple comparisons were used to analyse the data in GraphPad Prism 8.0. The data are shown as means ± standard deviations (S.D.). A P-value less than 0.05 was considered statistically significant (* P < 0.05, ** P < 0.01, *** P < 0.001).

## Results

### Selection of M2e epitope of M2 protein in influenza virus

**Fig. 1:**
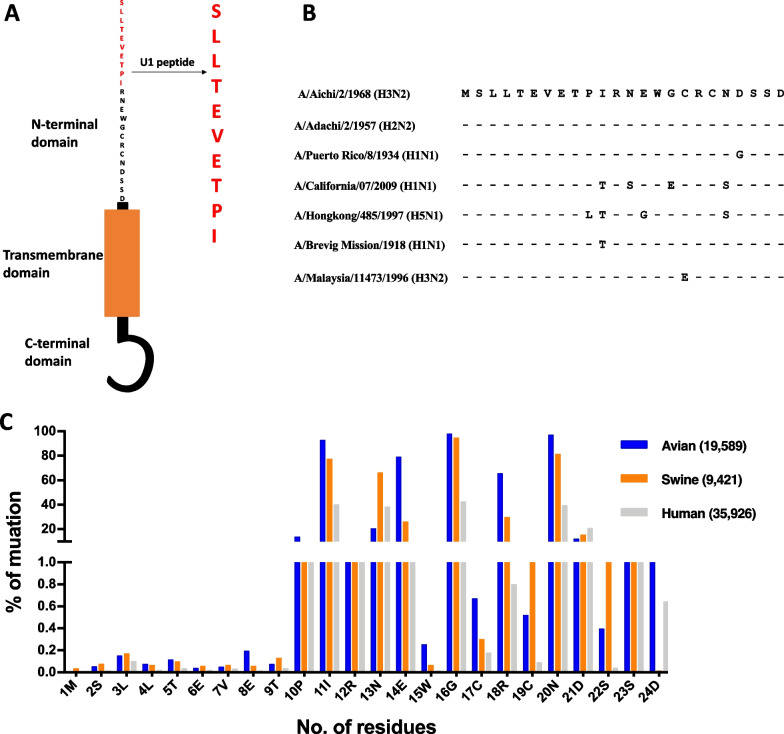
Selection of specific scFv phages against the conserved U1 (SLLTEVETPI) epitope M2e of influenza. **A** Schematic diagram of M2 protein of the influenza virus and U1 N-terminal conserved sequence highlighted in red. **B** Sequence alignment of M2e of M2 protein of different strains of the influenza viruses. **C** Percentage variations of M2e ectodomain at each residue. All strain sequences (64,936) were extracted from Influenza Research Database, and percentage mutations for each residue were calculated (http://www.fludb.org). (Y axis: Number of residues or amino acid residues in the sequence of M2e domain and X axis: Percentage of mutation per residue)

The M2 protein of the influenza virus has three domains (Fig. 1A). One of the domains is the N-terminal ectodomain (M2e) which is surface exposed, and suitable to interact with the antibodies. We compared the sequence of the N-terminal M2e domain (1–24) of the A/Aichi/2/1968 (H3N2) strain with the other strains of influenza viruses. The first 10 residues of M2e are almost 98 % conserved. Subsequently, we analyzed 64,936 sequences (Avian, Swine, and Human) obtained from the influenza research database (http://www.fludb.org) and calculated the percentage of mutations per residue of the M2e domain (Fig. 1C). The mutational rate in the first 10 amino acid residues of M2e is significantly low compared to the rest of the M2e residues in all strains. Owing to the conservation of the N-terminal MSLLTEVETP epitope, we considered it an appropriate target, the antibody against which could inhibit various strains of influenza viruses in humans. We selected a 10-mer conserved epitope of the N-terminal region of the M2 ectodomain as SLLTEVETPI (labeled U1) used for screening against the Tomlinson (I + J) scFv phage libraries.

### Screening of M2e peptide against naive human antibody libraries

**Fig. 2:**
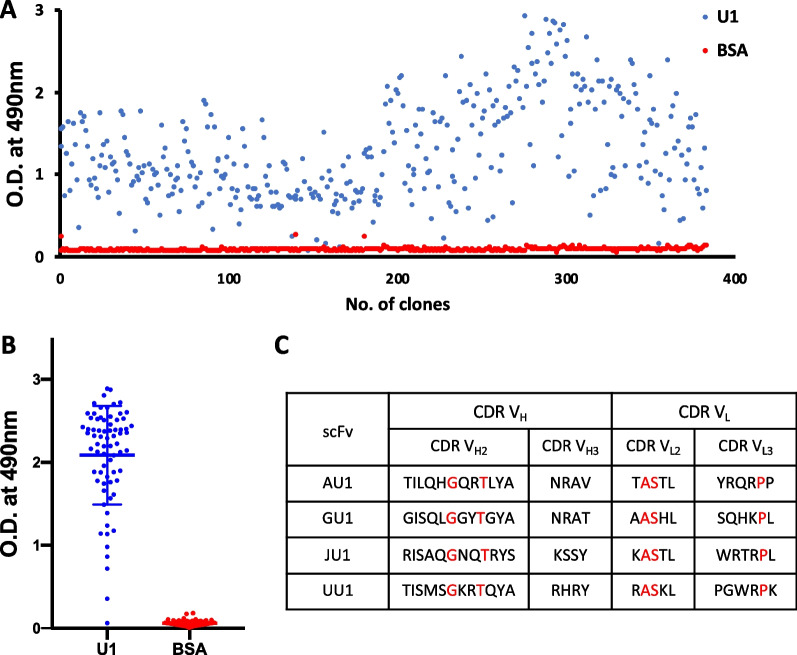
Screening of scFv against the U1 epitope. **A** In the fourth round of biopanning, approximately 400 individual scFv phages were selected, and the monoclonal phage ELISA was performed. Based on absorbance cutoff (O.D._490 nm_= 2.0), 72 high binder scFv phages were selected, and each phage was quantified. **B** After quantification of scFv phages, we picked phages in equal numbers (1 × 10^9^) to perform the monoclonal phage ELISA. The top 24 scFv phages were selected based on absorbance cutoff (O.D._490 nm_ = 2.4). **C** Sequencing of all scFvs revealed only 4 unique clones with sequence diversity in CDRs. Residues highlighted in red color are the same in all scFvs. All experiments were done in triplicate. The error bars represent standard deviations

The U1 peptide was synthesized with an additional lysine residue at the N-terminal end for conjugation with BSA, for screening purposes. The variable region of antibody is crucial for binding with the epitope and plays a significant role in viral inhibition. Therefore, we used scFv phage display library. Tomlinson (I + J) scFv phage libraries were screened against the BSA-conjugated U1 peptide. Four successive rounds of biopanning were carried out to enrich the specific scFv phages against BSA conjugated U1 peptide. At each successive round, polyclonal phage ELISA were carried out with scFv phages against the BSA conjugated U1 peptide and BSA as negative control resulting in affinity enrichment of scFv phage clones against the U1 peptide (Additional file 1: Fig. S1). The 384 individual phages were selected from the fourth round of biopanning after further enrichment and monoclonal phage ELISA of each phage clone from the bacterial supernatant was carried out (Fig. 2A). Varying affinity of scFv phages was observed against the antigen in the monoclonal phage ELISA. Seventy-two strong binders above O.D._490 nm_ value 2.0 were identified. These were further narrowed down to 24 phage clones after performing additional monoclonal ELISA against the antigen using purified and quantified phages (Fig. 2B). All 24 selected clones were sequenced, and multiple sequence alignment tool was used to examine the scFv sequences [21]. Four distinct clones (AU1, GU1, JU1, and UU1) were obtained based on changes in the CDR regions. Sequence variations in the clones were mostly limited to the CDRH2, CDRH3, CDRL2, and CDRL3 regions (Fig. 2C, Additional file 1: Fig. S2). The CDRH2 of each clone contained an amber stop codon (TAG), which is expressed as glutamine in the amber suppressor *E. coli* strain (Additional file 1: Fig. S2). To express functional scFvs in BL21(DE3) strain, site-directed mutagenesis was carried out to change amber stop codon (TAG) to glutamine (CAG) in the CDRH2 region of all four scFvs.

### Binding analysis and kinetics of scFvs with U1 peptide of M2e

**Fig. 3:**
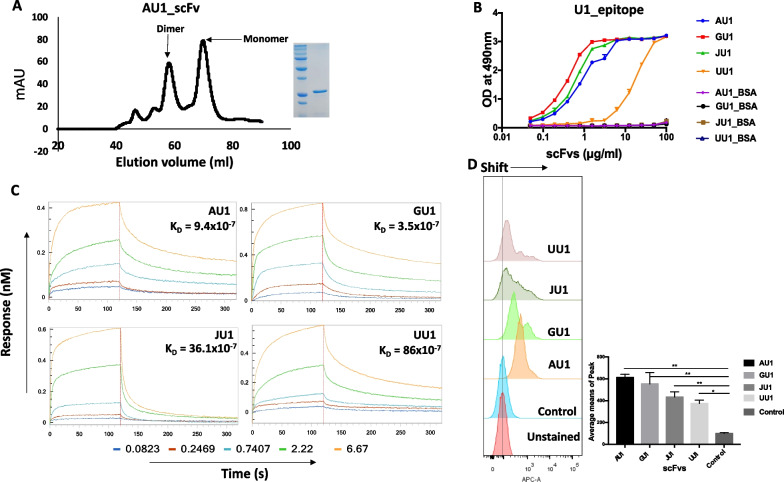
Binding analysis of purified scFvs.** A** Gel filtration chromatography profile of AU1. Monomer forms of scFvs were eluted at around 75 ml. Inset is the 12 % SDS-PAGE profile with purified scFv. **B** Direct ELISA with four selected purified scFvs against BSA conjugated U1 peptide with two-fold serial dilutions. Only scFv UU1 showed weak binding with peptide compared to other scFvs. **C** Bio-layer interferometry was carried out with all scFvs at different concentrations. Colors represent different concentrations that were serially diluted up to three times. All scFvs have a binding affinity (K_D_) in the sub-micromolar range. **D** Stable expression of native M2 protein on the surface of HEK293T cells was carried out, and its binding with the scFvs was performed by flow cytometry analysis. All scFvs showed a positive shift as compared to the control. The graph represents the average mean of the peaks. All experiments were performed in triplicate. Statistical analysis was performed using the one-way analysis of variance (ANOVA) with Bonferroni’s correction for multiple comparisons. ** P < 0.01; * P < 0.05. Data points represent averages of triplicates, and error bars represent standard deviations

All four unique scFvs were purified for the binding studies using affinity and gel filtration chromatography (Fig. 3A, Additional file 1: Fig. S3). In gel filtration chromatography, the peaks corresponding to the monomeric form of scFvs were used to perform all the ELISA and binding kinetics studies. We carried out a qualitative binding assay of purified scFvs against BSA-conjugated U1 peptide. The scFvs AU1, GU1, and JU1 demonstrated a high binding response with U1 peptide in ELISA compared to UU1 (Fig. 3B). We further carried out quantitative BLI kinetics assay to calculate the binding affinity of scFvs with U1 peptide. All scFvs demonstrated binding in the sub-micromolar affinity range (Fig. 3C). To further investigate if these four scFvs can also bind with the full-length M2 protein, we expressed the full-length M2 protein in HEK293T cell line and performed the flow cytometric assays. Distinct positive shift of the peak with respect to the control was seen in case of all the four scFvs indicating the interaction of scFvs with the full-length M2 protein expressed on HEK293T cells (Fig. 3D). Thus, the four scFvs could bind to the U1 peptide as well as M2 protein expressed in HEK293T cell line.

### The binding of U1 peptide dimer at the interface of two monomers of scFv AU1

We carried out crystallographic analysis of U1 complexed with scFvs. The crystallization experiments of all scFvs in apo form and with the peptide epitope at different molar ratios were carried out. However, the crystals of scFvs bound to U1 peptide were not observed. To facilitate crystallization, an extended U1 peptide with five additional amino acids were used during the process to improve scFv-peptide complex crystals. The crystals of scFv AU1 in the apo form and as a complex with the extended U1 peptide were obtained. Freshly purified apo scFv AU1 (8 mg/ml) in 20 mM Tris pH8.0, 50 mM NaCl was crystallized in 4 M sodium formate. The complex of scFv AU1 and the extended U1 peptide epitope was successfully crystallized using 0.2 M ammonium citrate dibasic buffer at pH 5.2 containing 20 % PEG 1000 at 20 °C. The diffraction data were collected at 2.8 and 3.4 Å resolutions for apo form and scFv AU1 in complex with extended U1 peptide, respectively (Additional file 1: Table S1). The scFv GU1 was crystallized in 4 % MPD, 0.5 M sodium citrate at pH 3.5, 20 % PEG 1500 in the apo form for which the data were collected at a resolution of 1.9 Å resolution (Additional file 1: Table S1).

**Fig. 4:**
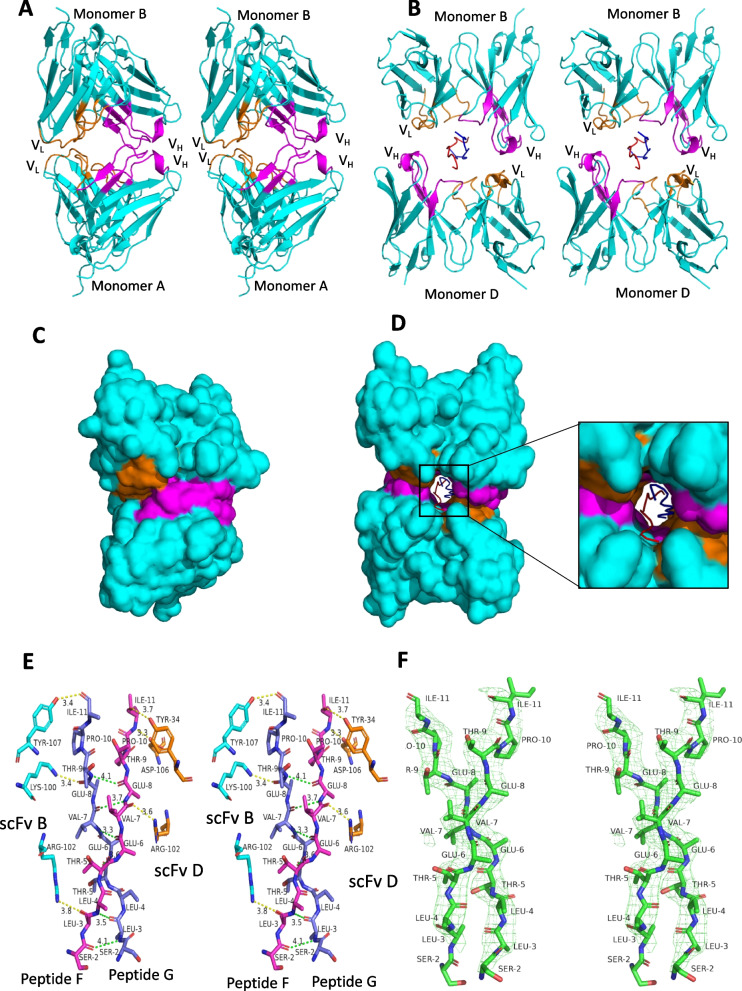
Structural comparison of scFv apo form and in complex with U1 peptide.** A** Stereo image of the apo structure of scFv AU1 depicting two molecules in the asymmetric unit. The CDR regions, variable heavy chain (V_H_) and variable light chain (V_L_), are shown in magenta and orange, respectively. **B** Stereo image of scFv AU1 in complex with extended U1 peptide in cartoon representation. Peptide monomers (F and G) are shown in red and blue colors. **C**, **D** Surface representation of scFv AU1 apo form and in complex with extended U1 peptide. The two peptide molecules snugly fit in a cavity between the CDR regions of the scFv molecule. **E** Interactions of the extended U1 peptide with scFv AU1 are shown in stereo mode. **F** Fo-Fc omit map of peptide dimer. The side chains of some residues were not modeled due to poor electron density

Apo form of scFv AU1, crystallized in the *P*4_2_2_1_2 space group with two molecules of scFv in the asymmetric unit. The co-crystals of scFv AU1 with extended U1 peptide crystallized in the *P*2_1_2_1_2_1_ space group with five molecules of scFv in the asymmetric unit (Additional file 1: Fig. S4). Of the five scFv molecules, three are unliganded, and organized as one dimer and one monomer. The remaining two molecules exist as a dimer with a peptide dimer at scFv dimeric interface. The two monomers of scFv in the unliganded dimer are related through a pseudo-two-fold axis with identical CDRs facing each other (Fig. 4A, Additional file 1: Fig. S5). In the co-crystals, the two liganded molecules of five scFvs are also related to each other through a two-fold axis. However, the axis is almost perpendicular to that observed in the apo structure resulting in CDRs of V_H_ facing CDRs of V_L_ (Additional file 1: Fig. S5). Distinct CDR orientations in liganded scFvs allows accommodation of the peptide at scFv interface in the form of a wider groove with extensive scFv-peptide interactions (Fig. 4B). A similar groove is absent in the apo form (Fig. 4C, D).

Interestingly the two peptide molecules are present in parallel dimer orientation at the interface of the two scFv molecules. They were stabilized by backbone hydrogen bonds providing a twisted parallel beta-sheet conformation to the peptide dimer (Fig. 4E). One molecule of scFv used interactions through the heavy chain, and another molecule of scFv used interactions through the light chain, making a stable complex with the peptide dimer. The residues Lys100 (CDR V_H3_), Arg102 (CDR V_H3_), and Tyr107 (CDR V_H3_) of scFv (monomer B) formed hydrogen bonds with both the peptide molecules. The other monomer of the scFv light chain also had significant hydrogen bonding interactions with one of the two peptide molecules (Fig. 4E). Eleven amino acids of the extended U1 peptide were modelled based on the Fo–Fc map contoured at 3σ cut-off (Fig. 4F).

A comparative structural analysis of liganded scFv AU1 with unliganded scFvs AU1 and GU1 and models of scFvs JU1 and UU1 was carried out. All the scFvs superimpose with RMSD ranging from 0.358 Å to 0.498 Å. Interestingly, no significant conformational differences could be observed in CDR conformations. Interestingly, if all the three scFvs (i.e., GU1, JU1, and UU1) were superimposed over liganded scFv AU1 homodimer, it was observed that scFv AU1 homodimer in the liganded state is stabilized by more molecular interactions compared to the other three scFvs, perhaps translating into functional consequences.

### The molecular dynamics of U1 peptide with scFv complex indicated a stable structural state

Structural analysis of scFv bound to a peptide epitope of M2e showed binding of the ectodomain in dimeric form and explained how scFv could hold the domain in a particular state. The complex of scFv AU1 and U1 peptide, as observed in the crystal structure, was subjected to the MD simulation after energy minimization in order to analyze the extent of conformational variations of U1 peptide and its interaction in the dimeric state in antibody bound form. The RMSD plot of scFv AU1 and its bound U1 peptide suggests a stable state with some fluctuations throughout the trajectory (Additional file 1: Figs. S6A, S7). Similarly, the peptide dimer remained stable at the interface of scFv AU1 monomers (Additional file 1: Fig. S6B). The RMSF (root mean square fluctuations) between the monomers of scFv AU1 followed an almost similar pattern (Additional file 1: Fig. S6C). In the case of peptide dimer, both peptide molecules had comparable RMSFs (Additional file 1: Fig. S6D). The interactions with scFv CDRs along with extensive network of hydrogen bonds between the peptide backbones contributed toward the stability of the M2e peptide in the dimeric form.

**Fig. 5:**
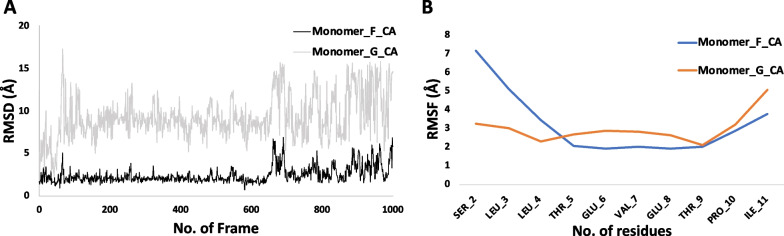
MD of monomer G and F without any scFv constraints.** A** RMSD of the peptide monomer of G and F without scFv for 500 ns. Both peptide molecules form internal backbone hydrogen bonds during the MD simulation of the trajectory. **B** RMSF of the peptides was stable between the residues. However, N-terminal was unstable during the trajectory

The peptide dimer, as found in crystal structure, was also subjected to MD simulation separately in order to evaluate its stability in the absence of any antibody. During the initial stages of MD simulation, the peptide dimer showed high fluctuations in RMSD values. After 50 ns, the U1 peptide remained stable throughout the trajectory (Fig. 5A, Additional file 1: Fig. S8). RMSF of both U1 peptide molecules demonstrated stability around Glu5 and Glu7 (Fig. 5B). The U1 peptide dimer indeed remained stable throughout the simulation.

### The scFv AU1 restricts the release of pdmH1N12009 viruses

We demonstrated that all four scFvs could bind to the fully expressed M2 protein of the influenza A virus in the HEK293T cell line. To check whether these four scFvs have the potential to inhibit influenza A virus replication, we screened the inhibition by all the scFvs in A549 cells infected with influenza A virus strain pdmH1N12009. Cells were incubated with each of the scFvs (100 µg/ml) for 8 h post viral adsorption and quantified the amount of viral RNA (vRNA) encoding pdmH1N12009 nucleoproteins in the supernatant (as the mature virions are released into the medium through budding) and in the infected cells (intracellular viruses) after single-round replication using quantitative real-time PCR.

**Fig. 6:**
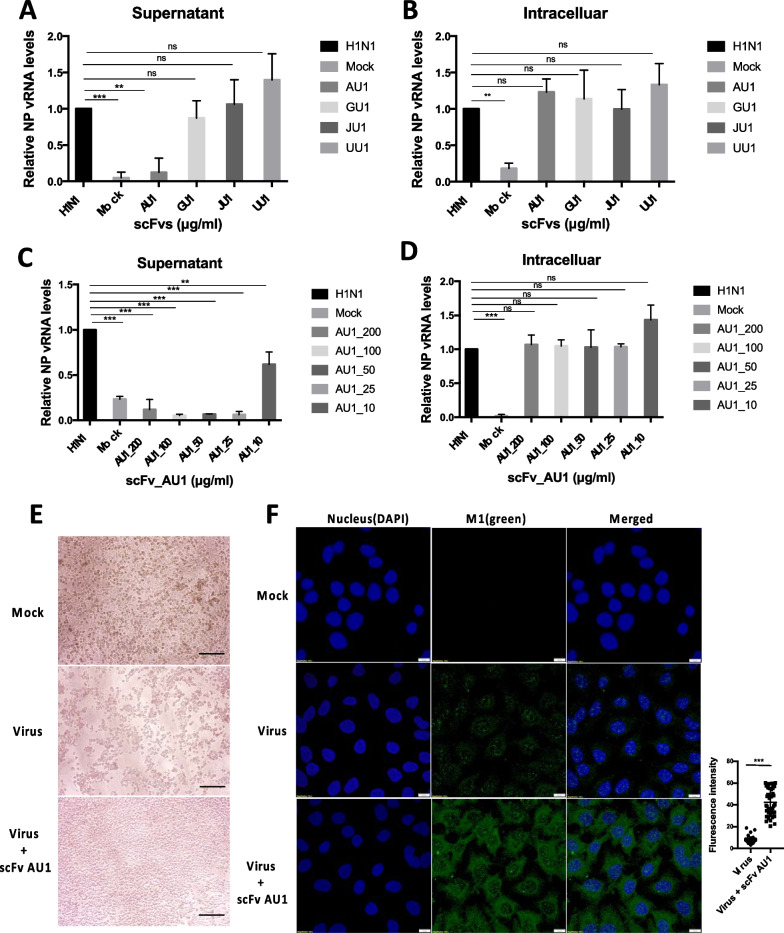
AU1 scFv restrict the growth of the pdmH1N12009 virus. A549 cells were infected with pdmH1N12009 at MOI 1.0 and fed replete medium supplemented with different scFv (100 µg/ml). NP vRNA was quantified after 8 h by RT PCR analysis. The values were normalized with GAPDH mRNA levels. **A** NP transcript level was measured in the supernatant of the A549 cell culture. Only scFv AU1 shows the restricted release of viruses. **B** The intracellular level of the NP gene is not affected significantly in infected cells by any of the scFvs. **C** scFv AU1 shows inhibition in a dose-dependent manner. NP vRNA level was significantly reduced at 25 µg/ml. **D** scFv AU1 did not show a significant reduction of NP level intracellularly. **E** Cytopathogenic effect of influenza virus in the presence of scFv AU1 was observed. In the mock, cells were healthy. In the presence of viral infection with H1N1 in A549, it showed significant cells death. In the presence of scFv AU1 with H1N1 virus infection in A549 cells, it showed the significant decreases of cells death. The morphological changes of A549 cells at 48 h post infection under a phase contrast inverted microscope were shown. Scale bars, 100 μm. **F** Immunofluorescence assay showed the higher viral load in infected cells in the presence of AU1 scFv compared to mock. The viral load is represented by the M1 protein (green). The DAPI blue stain represents the nucleus of a cell. Scale bars, 15 μm. Graph showing the quantification of fluorescence intensity. (N = 40) “N” indicates the number of cells analyzed. All experiments were performed in triplicate. Statistical analysis was performed using the one-way analysis of variance (ANOVA) with Bonferroni’s correction for multiple comparisons and students’ t-test. *** P < 0.001; ** P < 0.01; * P < 0.05. Data points represent averages of triplicates, and error bars represent standard deviations

It was observed that about 80 % reduction of the vRNA in the supernatant of pdmH1N12009-infected A549 cells treated with scFv AU1 compared to untreated pdmH1N12009-infected A549 cells (Fig. 6A). Intracellularly, there was no significant difference observed at viral gene levels with and without scFv treatment (Fig. 6B). It was noteworthy that after treatment with scFv AU1, the virus was able to enter and replicate inside the cell, which was confirmed by the transcript levels of nucleoprotein expression. However, addition of the scFvs GU1, JU1, and UU1, did not exhibit any significant difference between the H1N1 viral load with respect to untreated cells intracellularly as well as in the supernatant.

The antiviral effect of scFv AU1 against H1N1 influenza A virus is dose-dependent as 80 % inhibition at 25 µg/ml and 40 % inhibition at 10 µg/ml was observed (Fig. 6C). However, we did not see any significant reduction of viral load intracellularly (Fig. 6D). The virus-induced CPE was visualized under the microscope in order to check the effect of scFv AU1 on the infected cells. Complete lysis of infected cells, implying 100 % death, was noted in the H1N1 virus-infected cells in 48 h. However, in the presence of scFv AU1, no significant change in morphology was seen in the infected cells (Fig. 6E). In other words, scFv AU1 showed protection of cells, against the H1N1 virus.

We performed immunofluorescence assay to visualize the expression of the M1 viral protein in the infected cells in order to confirm the viral load at the protein level. Interestingly, it was observed that viral load in the presence of scFv AU1 was significantly higher than in the control (Fig. 6F). Apparently, virus could not be released from the infected cells into the extracellular environment in the presence of scFv AU1. It was anticipated that scFv AU1 limits viral release from infected cells in comparison to untreated scFv-infected cells.

## Discussion

The influenza A virus M2 protein is essential for virus propagation. It is crucial for breaking down the viral coat upon entry into the host cell and is needed for assembly and budding when viruses are released. The N-terminal ectodomain of the M2 ion channel is conserved among all influenza virus strains. It is considered a potential therapeutic target for designing universal vaccines. The N-terminal ten residues (MSLLTEVETP) in the M2 ion channel share common ORF with the M1 protein. The highly conserved nature may relate to functional attributes, and hence it is an attractive target for universal vaccine design [8, 15, 16, 31]. Some antibodies against the M2e region have potential to provides protection in vivo as well as inhibit virus at cellular level [17, 32]. The protection in vivo is provided by immune cells recruited via the antibody Fc region is well known. However, the mechanism of viral inhibition at cellular level is not completely elucidated at structure level. Thus, four unique antibody scFvs were obtained after the screening of the antibody scFv library against the U1 peptide epitope from the M2e domain. All four scFvs show physiological binding affinities toward the selected epitope and the M2 protein expressed on HEK293T cells indicating that these scFvs were specific and targeted against the conserved region of M2. The scFvs have several advantages and are used as therapeutic tools as they, besides giving high yield, also show a similar type of binding affinity as that of the whole antibodies towards the antigen [33].

**Fig. 7:**
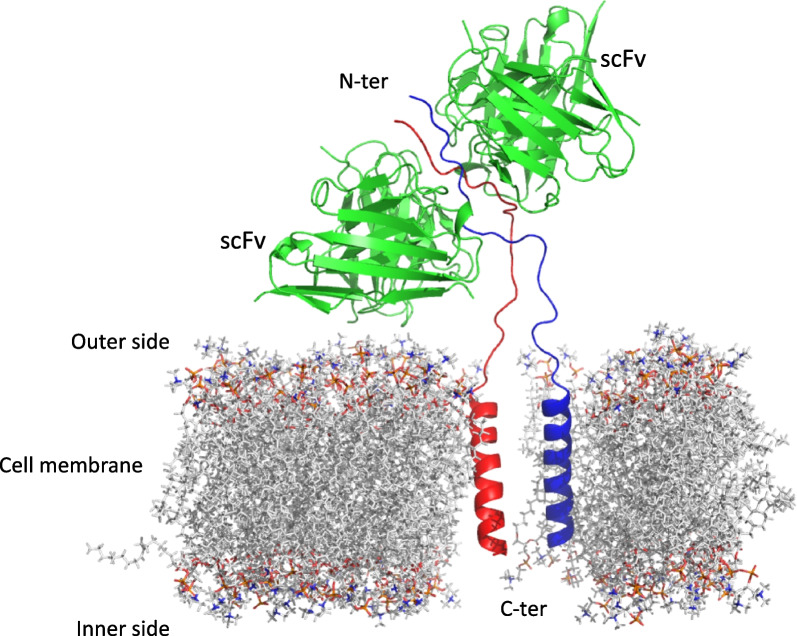
Schematic representation of M2 ion channel with scFvs at the structural level. Cartoon representation of the M2 protein in dimer form within the membrane and its interaction with scFv, guided by the structure of scFv-M2e peptide complex (PDB:8H3C)

Structural analysis of the unliganded scFv AU1 and that in complex with N-terminal M2e peptide epitope provided interesting insights. The peptide in dimeric form was bound between the antigen-combining grooves of two scFv monomers. The scFv AU1 residues also clasp both peptide molecules with hydrogen bonding interactions within the groove. Apart from the interactions of the M2e peptide with each scFvs, the two molecules of the peptide dimer sandwiched between the two scFv monomers had interactions within them, adapting a parallel ß-sheet conformation in the crystal structure. Thus, the two scFvs capture the peptide epitope in a dimeric parallel ß-sheet conformation perhaps trapping the entire membrane bound M2 protein in dimeric form with physiological consequences (Fig. 7). The peptide dimer could also exist in the absence of scFvs, as evidenced by the stability of this dimer during MD simulation. Other studies have also indicated that antibody binding to the M2e region could be in the dimeric form [20].

It could be inferred that in the presence of scFv AU1, only a limited release of virus progeny was observed into the medium by the cells infected with the pdmH1N12009 virus since a very low amount of viral gene transcripts in the supernatant were observed. Interestingly, the other three scFvs, did not show reduction of viral gene transcripts in the medium, although they bind to the M2 protein with comparable affinities. It is plausible that mode of interaction of scFv AU1 is distinct leading to M2 protein dimerization, which is not the case with other scFvs. It was also observed that virus-infected cells that were treated with scFv AU1 did not show any significant cytopathic effects. In the cytopathic assay, influenza virus-infected cells without scFv treatment showed complete loss of cell adhesion. They were floating in the medium indicating cell death. However, in the presence of scFv AU1, infected cells remained attached to the culture dish surface, suggesting the preservation of cell viability. This indicated that scFv AU1 was involved in protecting the cells from cytopathic effects induced by viral infection. Besides, the viral load with respect to M1 protein in the virus-infected cells was significantly high due to the presence of scFv AU1. Therefore, it is likely that the mature virus particles were retained in the cell. Thus, the scFv AU1 restricted the virus release from the infected cells but the molecular mechanism was not clear. Therefore, we believe that the anti-M2e scFvs could block the assembly of viruses. It has been reported that M2 proteins are involved in budding off of the viruses in the filamentous form from the infected cells [34, 35]. Earlier reported M2-specific antibodies showed inhibition of the filamentous form of the virion as well as disturbed the even distribution of expressed M2 protein on the host cell membrane [36, 37].

M2 protein is functional in the oligomeric form [14], and many anti-M2e antibodies do not inhibit viral replication due to their inability to alter the M2 functional complex. Evidences suggest that the M2 protein in tetrameric has all the structural elements to form a functional proton channel with four transmembrane helices forming a well-defined pore [38]. Assembly of M2 as a tetramer is a dynamic process involving monomer-to-dimer formation followed by dimer-to-tetramer transition [39]. Interestingly, our results showed that scFv AU1 could stop newly formed virus release from the infected cells while interacting with the N-terminal conserved region of M2 in a dimeric state. It is possible that antibody scFvs, other than AU1 do not form similar dimeric interactions with the epitope and that could explain their inability to inactivate the virus. The structural data as well as cell-based assays indicate that scFv AU1 has the capacity to alter the M2 protein functional complex possibly by inhibiting the formation of M2 tetramer.

## Conclusions

In conclusion, scFv AU1 can restrict assembly of the viral components of the influenza A virus H1N1 pandemic strain from the host cell possibly by altering the functional state of the M2 protein. This work opens a window to understand and engineer the universal therapeutic antibodies to neutralize diverse strains of influenza viruses due to conserved nature of the M2 protein. This work sets a platform to test engineered scFvs in animal-based models to establish possible protection in vivo by these scFvs.

## Supplementary information

**Additional file 1: Fig. S1.** Polyclonal phage ELISA. Polyclonal phages in the third and fourth rounds of biopanning showed the highest binding clones in ELISA when compared to the first and second rounds of biopanning. **Fig. S2.** Sequencing alignment analysis of the four unique clones against the U1 peptide. In the CDRs region of scFvs, Tom (I+J) libraries have a stop codon (TAG) that reads as glutamine amino acid in the TG-1 strain. For the expression in E. coli BL-21, we need to convert the stop codon (TAG) into glutamine amino acid codon (CAG) by changing a single nucleotide. The CDRH2 (highlighted with the small square box) has a stop codon mutated into the glutamine amino acid residues by site-directed mutagenesis. The large square box highlights other CDRs regions also. Sequencing alignment has been done by Clustal Omega (https://www.ebi.ac.uk/). **Fig. S3.** Purification profile of scFv GU1, scFv JU1 and scFv UU1. scFvs exist in both dimer and monomeric forms. We only collected the monomeric form of scFv and performed all experiments. **Fig. S4.** Co-crystal model of AU1 scFv with peptide. It shows five monomers of scFv in an asymmetric unit cell. **Fig. S5.**  Comparison of chains A and C (green) with chains B and D (cyan) in complex scFv AU1. (A) Chain B and D with dimer peptide of M2e (blue) in complex scFv AU1. (B) Chain A and C without dimer peptide of M2e in complex of scFv AU1. (C) Stereo image of the overlapping chains A and C model with chains B and D of scFv complex AU1. To entrap the peptide dimer between the groove of scFvs, the unliganded monomer has to rotate at 92.59^o^ and displaced at 15.71 Å. **Fig. S6.** Molecular dynamics studies of scFv AU1 in complex with U1 peptide.  (A) MD simulation of scFv and peptide complex was performed for 500ns. RMSDs (root mean square deviation) of the scFv and its epitope complex were relatively stable throughout the trajectory. (B) Stereo diagram representation of scFv AU1 complex snapshot at 100ns. (C) RMSF of the scFv monomers B and D. Both monomers had relatively similar fluctuations. (D) RMSF of the peptide monomers (G and F). **Fig. S7.** Molecular dynamics of scFv AU1 with peptide dimer. It showed the different time frames of the scFv AU1 with peptide dimer. Peptide dimer in between the groove of scFv AU1 was stable throughout the MD trajectory for500ns. **Fig. S8.** Molecular dynamics of the peptide. It shows the different time frames of the peptide dimer without scFv AU1. Peptide dimer was stable throughout the trajectory of MD for 500ns. **Table S1.** Data collection and refinement statistics.

## Data Availability

The atomic coordinates and structure factors have been deposited in the Protein Data Bank, http://www.wwpdb.org (PDB ID code: 8H3B, 8H3C, 8H73).

## References

[1] Monto AS (1987). Influenza. Quantifying morbidity and mortality. Am J Med..

[2] Petrova VN, Russell CA (2018). The evolution of seasonal influenza viruses. Nat Rev Microbiol.

[3] Steel J, Lowen AC (2014). Influenza a virus reassortment. Curr Top Microbiol Immunol.

[4] Harrington WN, Kackos CM, Webby RJ (2021). The evolution and future of influenza pandemic preparedness. Exp Mol Med.

[5] Fiore AE, Bridges CB, Cox NJ (2009). Seasonal influenza vaccines. Curr Top Microbiol Immunol.

[6] Krammer F, Palese P (2015). Advances in the development of influenza virus vaccines. Nat Rev Drug Discov.

[7] Hu L, Lao G, Liu R, Feng J, Long F, Peng T (2023). The race toward a universal influenza vaccine: front runners and the future directions. Antiviral Res.

[8] Lamb RA, Lai CJ, Choppin PW (1981). Sequences of mRNAs derived from genome RNA segment 7 of influenza virus: colinear and interrupted mRNAs code for overlapping proteins. Proc Natl Acad Sci U S A.

[9] Lamb RA, Zebedee SL, Richardson CD (1985). Influenza virus M2 protein is an integral membrane protein expressed on the infected - cell surface. Cell.

[10] Helenius A (1992). Unpacking the incoming influenza virus. Cell.

[11] Leiding T, Wang J, Martinsson J, DeGrado WF, Arsköld SP (2010). Proton and cation transport activity of the M2 proton channel from influenza a virus. Proc Natl Acad Sci U S A.

[12] Kawano K, Yano Y, Matsuzaki K (2014). A dimer is the minimal proton - conducting unit of the influenza a virus M2 channel. J Mol Biol.

[13] Sugrue RJ, Hay AJ (1991). Structural characteristics of the M2 protein of influenza a viruses: evidence that it forms a tetrameric channel. Virology.

[14] Holsinger LJ, Lamb RA (1991). Influenza virus M2 integral membrane protein is a homotetramer stabilized by formation of disulfide bonds. Virology.

[15] Kolpe A, Schepens B, Fiers W, Saelens X (2017). M2 - based influenza vaccines: recent advances and clinical potential. Expert Rev Vaccines.

[16] Ito T, Gorman OT, Kawaoka Y, Bean WJ, Webster RG (1991). Evolutionary analysis of the influenza a virus M gene with comparison of the M1 and M2 proteins. J Virol.

[17] Zebedee SL, Lamb RA (1988). Influenza a virus M2 protein: monoclonal antibody restriction of virus growth and detection of M2 in virions. J Virol.

[18] Kim M - C, Song J - M, Kwon OE, Lee Y - M, Compans Y - J (2013). Virus - like particles containing multiple M2 extracellular domains confer improved cross - protection against various subtypes of influenza virus. Mol Ther J Am Soc Gene Ther.

[19] Neirynck S, Deroo T, Saelens X, Vanlandschoot P, Jou WM, Fiers W (1999). A universal influenza a vaccine based on the extracellular domain of the M2 protein. Nat Med.

[20] Fu T - M, Freed DC, Horton MS, Fan J, Citron MP, Joyce JG (2009). Characterizations of four monoclonal antibodies against M2 protein ectodomain of influenza a virus. Virology.

[21] Thompson JD, Higgins DG, Gibson TJ (1994). CLUSTAL W: improving the sensitivity of progressive multiple sequence alignment through sequence weighting, position-specific gap penalties and weight matrix choice. Nucleic Acids Res..

[22] Vashisht S, Verma S, Salunke DM (2019). Cross - clade antibody reactivity may attenuate the ability of influenza virus to evade the immune response. Mol Immunol.

[23] Rouet R, Lowe D, Dudgeon K, Roome B, Schofield P, Langley D (2012). Expression of high - affinity human antibody fragments in bacteria. Nat Protoc.

[24] Concepcion J, Witte K, Wartchow C, Choo S, Yao D, Persson H (2009). Label - free detection of biomolecular interactions using BioLayer interferometry for kinetic characterization. Comb Chem High Throughput Screen.

[25] Minor W, Cymborowski M, Otwinowski Z, Chruszcz M (2006). HKL - 3000: the integration of data reduction and structure solution–from diffraction images to an initial model in minutes. Acta Crystallogr D Biol Crystallogr.

[26] Vonrhein C, Flensburg C, Keller P, Sharff A, Smart O, Paciorek W (2011). Data processing and analysis with the autoPROC toolbox. Acta Crystallogr D Biol Crystallogr.

[27] Liebschner D, Afonine PV, Baker ML, Bunkóczi G, Chen VB, Croll TI (2019). Macromolecular structure determination using X - rays, neutrons and electrons: recent developments in Phenix. Acta Crystallogr Sect Struct Biol.

[28] Emsley P, Cowtan K (2004). Coot: model - building tools for molecular graphics. Acta Crystallogr D Biol Crystallogr.

[29] Bowers K, Chow E, Xu H, Dror R, Eastwood M, Gregersen B et al. Molecular dynamics—scalable algorithms for molecular dynamics simulations on commodity clusters. Supercomput. 2006 SC06 Proc. ACMEEEI. 2006.

[30] Cho KJ, Schepens B, Seok JH, Kim S, Roose K, Lee J - H (2015). Structure of the extracellular domain of matrix protein 2 of influenza a virus in complex with a protective monoclonal antibody. J Virol.

[31] Schotsaert M, De Filette M, Fiers W, Saelens X (2009). Universal M2 ectodomain - based influenza a vaccines: preclinical and clinical developments. Expert Rev Vaccines.

[32] Liu W, Zou P, Chen Y - H (2004). Monoclonal antibodies recognizing EVETPIRN epitope of influenza a virus M2 protein could protect mice from lethal influenza a virus challenge. Immunol Lett.

[33] Monnier PP, Vigouroux RJ, Tassew NG (2013). In Vivo Applications of Single Chain Fv (Variable Domain) (scFv) Fragments. Antibodies..

[34] Rossman JS, Jing X, Leser GP, Balannik V, Pinto LH, Lamb RA (2010). Influenza virus m2 ion channel protein is necessary for filamentous virion formation. J Virol.

[35] Rossman JS, Jing X, Leser GP, Lamb RA (2010). Influenza virus M2 protein mediates ESCRT - independent membrane scission. Cell.

[36] Manzoor R, Eguchi N, Yoshida R, Ozaki H, Kondoh T, Okuya K (2020). A novel mechanism underlying antiviral activity of an Influenza Virus M2 - Specific antibody. J Virol.

[37] Kolpe A, Arista - Romero M, Schepens B, Pujals S, Saelens X, Albertazzi L (2019). Super - resolution microscopy reveals significant impact of M2e - specific monoclonal antibodies on influenza a virus filament formation at the host cell surface. Sci Rep.

[38] Schnell JR, Chou JJ (2008). Structure and mechanism of the M2 proton channel of influenza a virus. Nature.

[39] Georgieva ER, Borbat PP, Norman HD, Freed JH (2015). Mechanism of influenza A M2 transmembrane domain assembly in lipid membranes. Sci Rep.

