# Supplementary material for: A structure and knowledge-based combinatorial approach to engineering universal scFv antibodies against influenza M2 protein
Source: J Biomed Sci. 2023 Jul 25;30:56. doi: 10.1186/s12929-023-00950-2 (PMC10367348; doi:10.1186/s12929-023-00950-2)
Supplement: Supplementary file 1 — Additional file 1: Fig. S1. Polyclonal phage ELISA. Polyclonal phages in the third and fourth rounds of biopanning showed the highest binding clones in ELISA when compared to the first and second rounds of biopanning. Fig. S2. Sequencing alignment analysis of the four unique clones against the U1 peptide. In the CDRs region of scFvs, Tom (I+J) libraries have a stop codon (TAG) that reads as glutamine amino acid in the TG-1 strain. For the expression in E. coli BL-21, we need to convert the stop codon (TAG) into glutamine amino acid codon (CAG) by changing a single nucleotide. The CDRH2 (highlighted with the small square box) has a stop codon mutated into the glutamine amino acid residues by site-directed mutagenesis. The large square box highlights other CDRs regions also. Sequencing alignment has been done by Clustal Omega (https://www.ebi.ac.uk/). Fig. S3. Purification profile of scFv GU1, scFv JU1 and scFv UU1. scFvs exist in both dimer and monomeric forms. We only collected the monomeric form of scFv and performed all experiments. Fig. S4. Co-crystal model of AU1 scFv with peptide. It shows five monomers of scFv in an asymmetric unit cell. Fig. S5. Comparison of chains A and C (green) with chains B and D (cyan) in complex scFv AU1. (A) Chain B and D with dimer peptide of M2e (blue) in complex scFv AU1. (B) Chain A and C without dimer peptide of M2e in complex of scFv AU1. (C) Stereo image of the overlapping chains A and C model with chains B and D of scFv complex AU1. To entrap the peptide dimer between the groove of scFvs, the unliganded monomer has to rotate at 92.59o and displaced at 15.71 Å. Fig. S6. Molecular dynamics studies of scFv AU1 in complex with U1 peptide. (A) MD simulation of scFv and peptide complex was performed for 500ns. RMSDs (root mean square deviation) of the scFv and its epitope complex were relatively stable throughout the trajectory. (B) Stereo diagram representation of scFv AU1 complex snapshot at 100ns. (C) RMSF of the scFv monomers B [file 12929_2023_950_MOESM1_ESM.docx]

**Additional Figures**


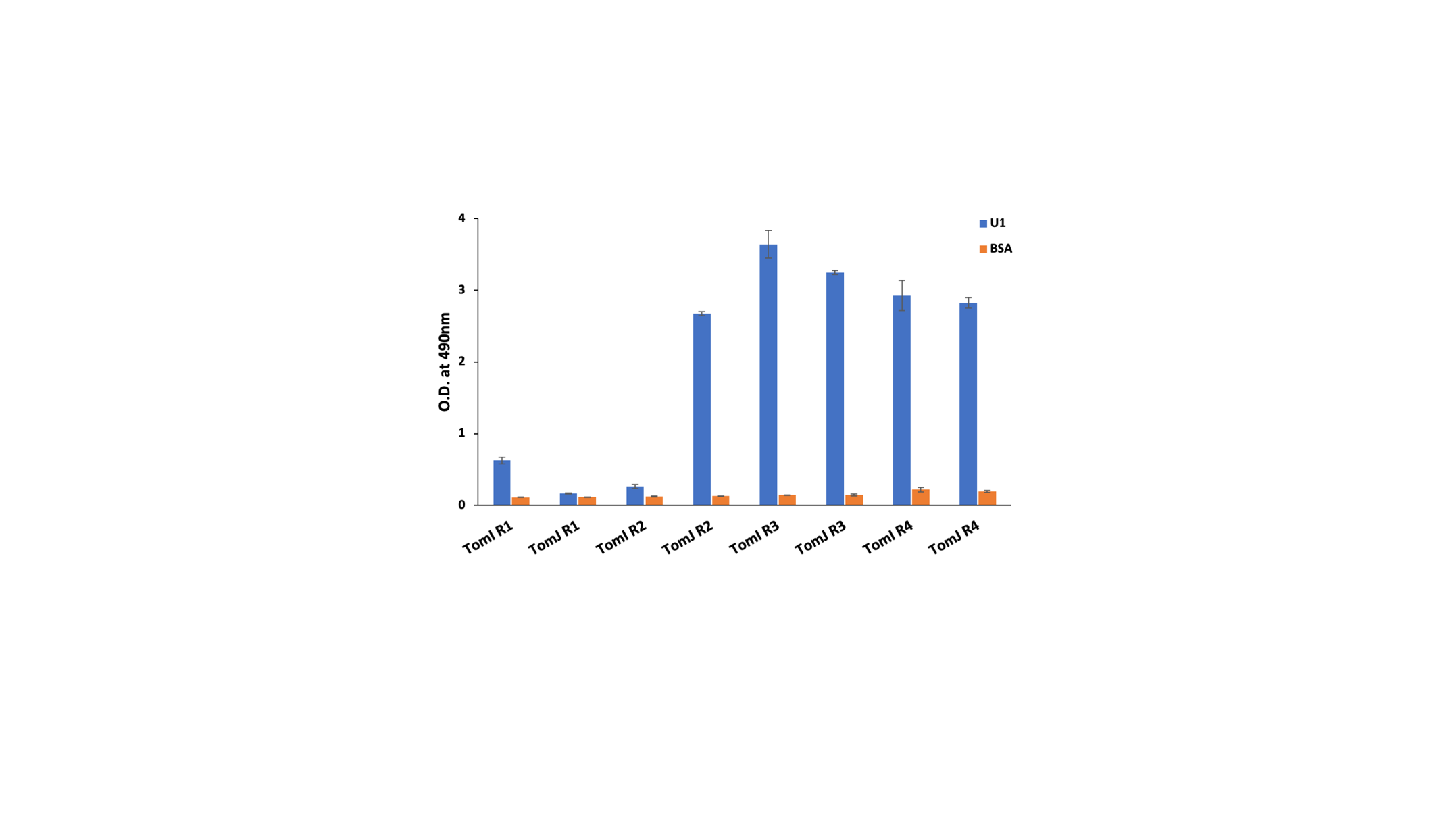


***Fig. S1. Polyclonal phage ELISA.*** *Polyclonal phages in the third and fourth rounds of biopanning showed the highest binding clones in ELISA when compared to the first and second rounds of biopanning.*

***Fig. S2.*** ***Sequencing alignment analysis of the four unique clones against the U1 peptide.*** *In the CDRs region of scFvs, Tom (I+J) libraries have a stop codon (TAG) that reads as glutamine amino acid in the TG-1 strain. For the expression in E. coli BL-21, we need to convert the stop codon (TAG) into glutamine amino acid codon (CAG) by changing a single nucleotide. The CDRH2 (highlighted with the small square box) has a stop codon mutated into the glutamine amino acid residues by site-directed mutagenesis. The large square box highlights other CDRs regions also. Sequencing alignment has been done by Clustal Omega (https://www.ebi.ac.uk/).*

***Fig. S3. Purification profile of scFv GU1, scFv JU1 and scFv UU1****. scFvs exist in both dimer and monomeric forms. We only collected the monomeric form of scFv and performed all experiments.*


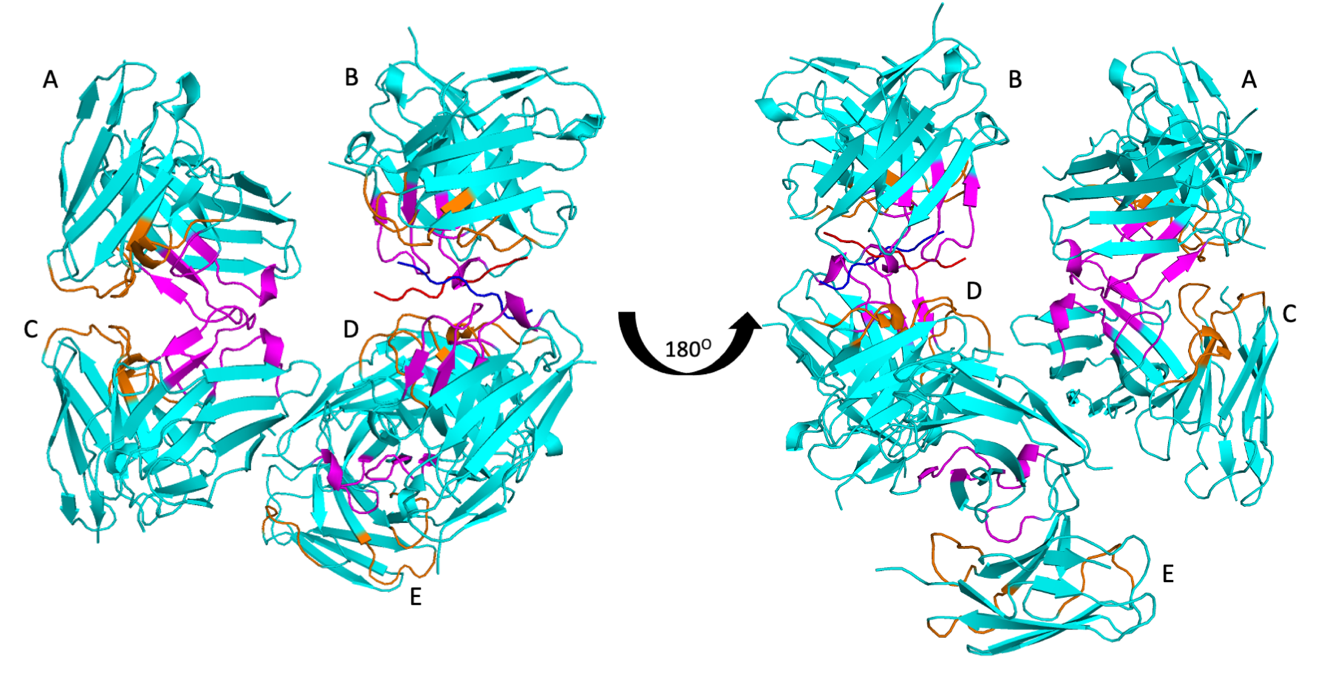


***Fig. S4. Co-crystal model of AU1 scFv with peptide.*** *It shows five monomers of scFv in an asymmetric unit cell.*


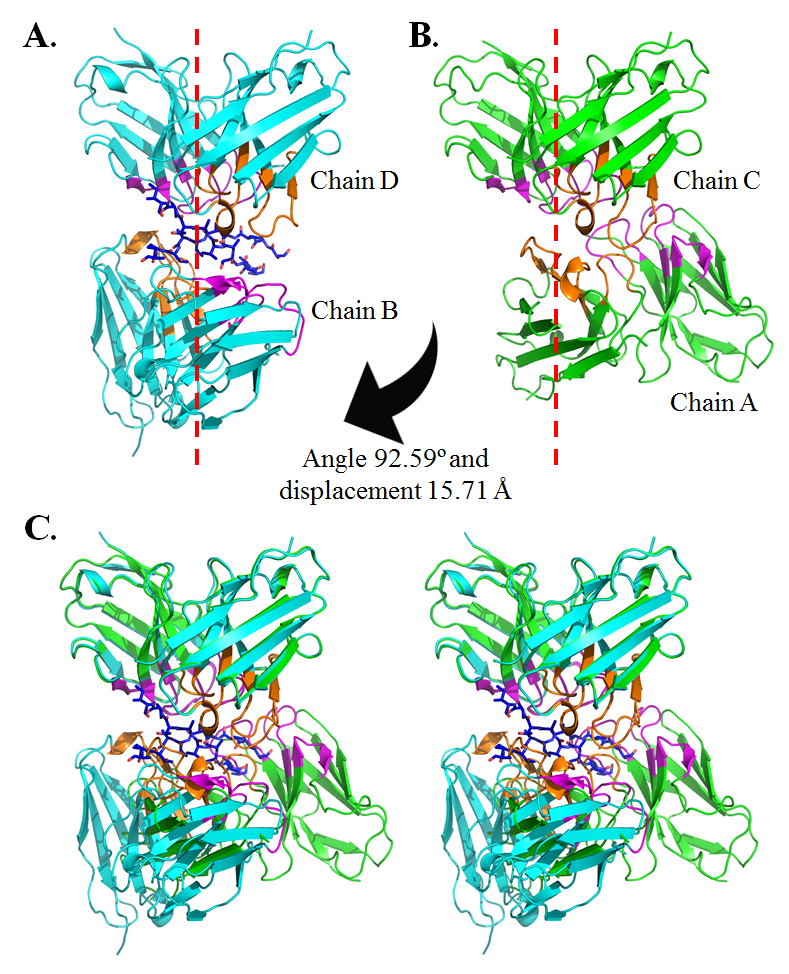


***Fig. S5. Comparison of chains A and C (green) with chains B and D (cyan) in complex scFv AU1****. (A) Chain B and D with dimer peptide of M2e (blue) in complex scFv AU1. (B) Chain A and C without dimer peptide of M2e in complex of scFv AU1. (C) Stereo image of the overlapping chains A and C model with chains B and D of scFv complex AU1. To entrap the peptide dimer between the groove of scFvs, the unliganded monomer has to rotate at 92.59^o^ and displaced at 15.71 Å.*

***Fig. S6.*** ***Molecular dynamics studies of scFv AU1 in complex with U1 peptide.*** *(A) MD simulation of scFv and peptide complex was performed for 500ns. RMSDs (root mean square deviation) of the scFv and its epitope complex were relatively stable throughout the trajectory. (B) Stereo diagram representation of scFv AU1 complex snapshot at 100ns. (C) RMSF of the scFv monomers B and D. Both monomers had relatively similar fluctuations. (D) RMSF of the peptide monomers (G and F).*


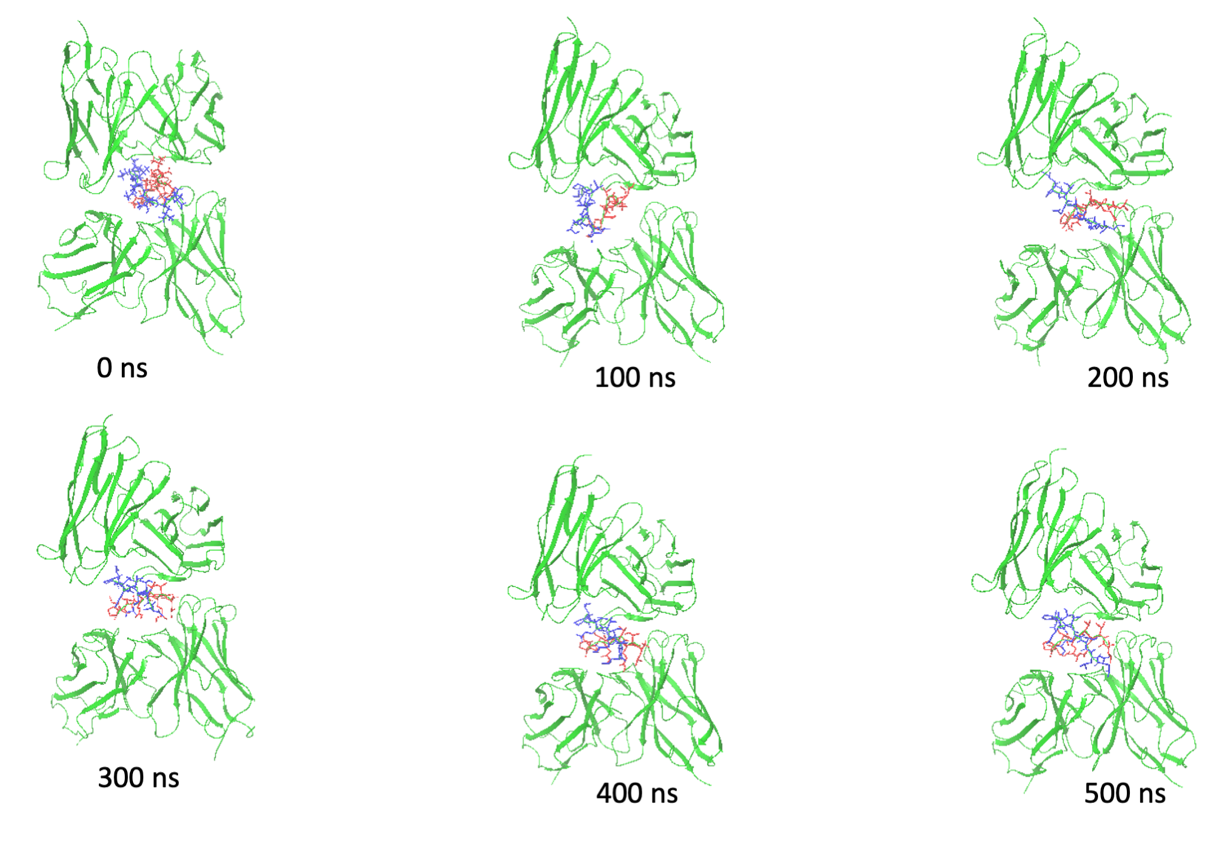


***Fig. 7. Molecular dynamics of scFv AU1 with peptide dimer****. It showed the different time frames of the scFv AU1 with peptide dimer. Peptide dimer in between the groove of scFv AU1 was stable throughout the MD trajectory for 500ns.*

***Fig. S8. Molecular dynamics of the peptide.*** *It shows the different time frames of the peptide dimer without scFv AU1. Peptide dimer was stable throughout the trajectory of MD for 500ns.*

**Table S1. Data collection and refinement statistics**

| **PDB ID** | 8H3B | 8H3C | 8H73 |
| --- | --- | --- | --- |
| **Diffraction statistics** |  |  |  |
| X-ray source | ID30B, ESRF | ID30B, ESRF | XRD2, ELETRA |
| Space Group | P4_2_2_1_2 | P2_1_2_1_2_1_ | P6_1_22 |
| Unit cell dimensions a,b,c (Å) α,β,γ (⁰) | 106.97, 106.97, 149.21  90, 90, 90 | 104.82,120.40,176.86  90, 90, 90 | 175.03,175.03,45.92  90, 90, 120 |
| Resolution (Å) | 75.64 - 2.75  (2.80 – 2.75) | 60.20 - 3.43  (3.49 – 3.43) | 43.95 - 1.91  (1.97 – 1.91) |
| R_merge_ (%) | 15.9 (86.9) | 25.0 (85.3) | 12.1 (76.5) |
| CC_1/2_ (%) | 99.5 (84.0) | 98.3 (68.5) | 99.9(69.4) |
| I/𝝈I | 12.9 (3.4) | 6.7 (2.1) | 18.2 (2.2) |
| Completeness (%) | 99.6 (98.7) | 99.7 (100) | 97.9 (94.5) |
| Redundancy | 10.0 (7.8) | 4.0 (3.9) | 12.8 (10.2) |
| **Refinement statistics** |  |  |  |
| Resolution (Å) | 75.64 - 2.75  (2.80 – 2.75) | 60.21 - 3.43  (3.55 – 3.43) | 40.67  - 1.91  (1.97 – 1.91) |
| No. of reflections | 23024 (2234) | 30599 (3039) | 31713 (2923) |
| R_work_/R_free_ | 0.1764 / 0.2051 | 0.2039/ 0.2347 | 0.1807/0.2010 |
| No. of atoms  Protein  Ligand/Ion  Water | 3482  3404  6  72 | 8520  8520  0  0 | 1865  1671  13  181 |
| Average B-factor (Å^2^)  Protein  Ligand/Ion  Water | 47.46  47.48  67.55  44.74 | 82.36  82.36  -  - | 28.66  27.37  41.58  39.60 |
| RMS deviations  Bond length(Å)  Bond angles (⁰) | 0.003  0.72 | 0.003  0.62 | 0.011  1.09 |
| Ramachandran statistics  Favoured (%)  Allowed (%)  Outliers (%) | 97.27  2.73  0.00 | 96.28  3.54  0.00 | 96.33  3.67  0.00 |
